# DNA-BOT: a low-cost, automated DNA assembly platform for synthetic biology

**DOI:** 10.1093/synbio/ysaa010

**Published:** 2020-07-09

**Authors:** Marko Storch, Matthew C Haines, Geoff S Baldwin

**Affiliations:** y1 Department of Life Sciences, Imperial College London, London, SW7 2AZ, UK; y2 Imperial College Centre for Synthetic Biology, Imperial College London, London, SW7 2AZ, UK; y3 London Biofoundry, Imperial College Translation & Innovation Hub, London, W12 0BZ, UK

**Keywords:** DNA assembly, automation, synthetic biology, biofoundry

## Abstract

Multi-part DNA assembly is the physical starting point for many projects in Synthetic and Molecular Biology. The ability to explore a genetic design space by building extensive libraries of DNA constructs is essential for creating programmed biological systems. With multiple DNA assembly methods and standards adopted in the Synthetic Biology community, automation of the DNA assembly process is now receiving serious attention. Automation will enable larger builds using less researcher time, while increasing the accessible design space. However, these benefits currently incur high costs for both equipment and consumables. Here, we address this limitation by introducing low-cost DNA assembly with BASIC on OpenTrons (DNA-BOT). For this purpose, we developed an open-source software package and demonstrated the performance of DNA-BOT by simultaneously assembling 88 constructs composed of 10 genetic parts, evaluating the promoter, ribosome binding site and gene order design space for a three-gene operon. All 88 constructs were assembled with high accuracy, at a consumables cost of $1.50–$5.50 per construct. This illustrates the efficiency, accuracy and affordability of DNA-BOT, making it accessible for most labs and democratizing automated DNA assembly.

## 1. Introduction

Creating DNA constructs is the foundational process that allows biologists to engineer and interrogate biological systems for a wide range of applications in basic research, biotechnology and more recently data storage (1, 2). Consequently, DNA assembly techniques and standards have evolved to address the desire to construct diverse sequences ranging in sizes from plasmids to whole genomes (1). As with many routine molecular biology methods, workflow standardization has enabled DNA assembly techniques to be completely automated, increasing the scale of construction and extending the addressable design space. This approach has now led to the emergence of Biofoundries (3).

**Figure 1.**
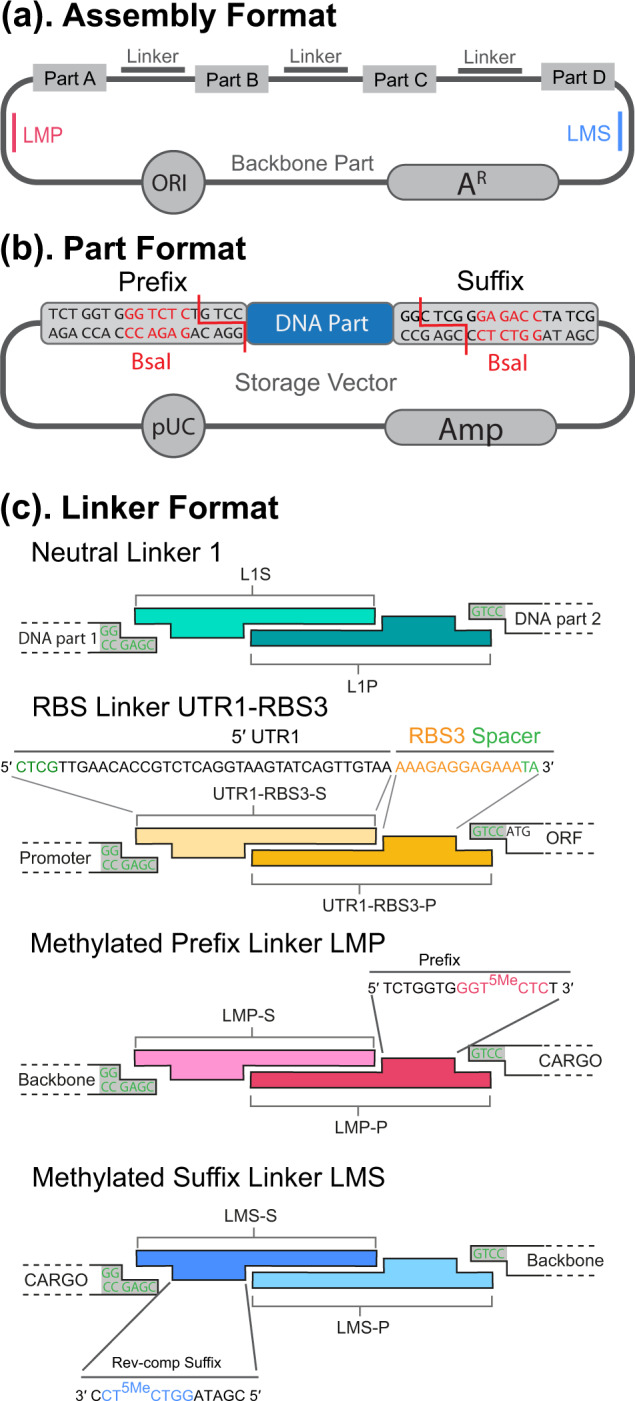
BASIC DNA assembly. (**a**) BASIC provides an assembly format where each DNA ‘part’ is joined with a linker. The parts of interest are assembled into a standard backbone, comprised of an origin of replication (ORI) and an antibiotic resistance marker (A^R^). The assembled parts are flanked by linkers that recapitulate the BASIC Prefix and Suffix (LMP and LMS), thus generating the construct in the idempotent BASIC standard. (**b**) DNA parts are usually stored in high-copy number ampicillin resistance vectors, flanked by the BASIC Prefix and Suffix sequences with BsaI restriction sites used to release the parts from the vectors. (**c**) Linkers are synthetic oligonucleotides annealed to form half-linkers with 4 bp overhangs that are specific for the Prefix and Suffix overhangs and 21 bp single-stranded overhangs that direct the assembly; each half-linker is ligated to specific parts in separate clip reactions. Linkers used in this study are either UTR-RBS linkers that encode an RBS within a defined 5′-UTR; or methylated Prefix and Suffix linkers; fusion linkers can also be used to create fusion proteins and neutral linkers are available with no defined function (4).

We previously developed the Biopart Assembly Standard for Idempotent Cloning (BASIC) method and standard to enable highly accurate multi-part DNA assembly at both manual bench and fully automated Biofoundry scale (4, 5). BASIC uses standard computationally designed linkers (6, 7) to join parts in a Part-Linker-Part-Linker-format (Figure 1a). The DNA parts are defined by a single storage format with prefix and suffix sequences flanking each part, usually stored in a high-copy vector (Figure 1b). The linkers can also function as composable parts, thus greatly enriching the design space: UTR-RBS linkers enable tuning of ribosome binding site (RBS) strength within a consistent 5′-untranslated region (UTR) (Figure 1c) and linkers with a complete coding sequence read through can be used to create fusion proteins (4). The linkers are physically split into two partially double-stranded half-linkers with each half being separately ligated to the Suffix and Prefix junctions of BsaI digested parts. In addition, the Prefix and Suffix sequence can be coded on linkers (LMP and LMS, respectively) by protecting the BsaI site from cleavage during assembly with a single C-5 methyl group (Figure 1c; (4)). This enables a single-tier idempotent assembly format; by flanking the assembled parts of interest with LMP and LMS linkers (Figure 1a), the new construct is returned in BASIC format and can be used as an input to further rounds of assembly.

**Figure 2.**
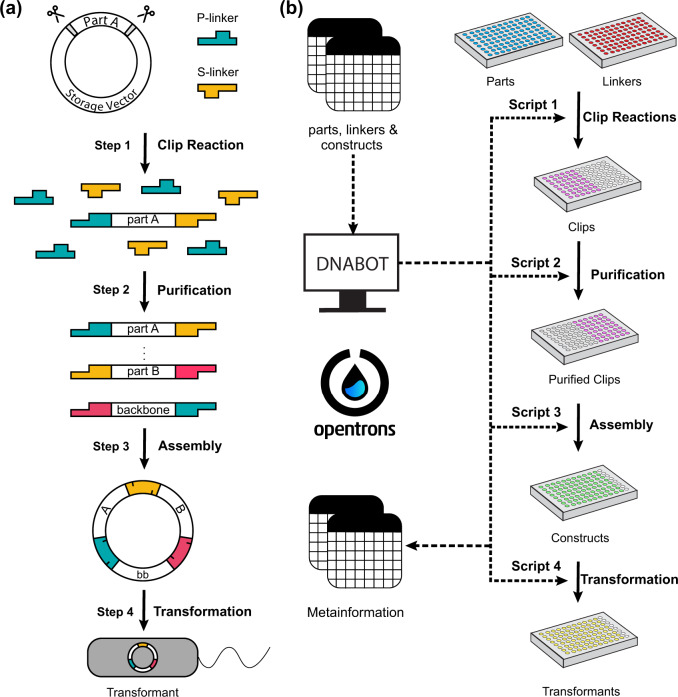
BASIC DNA assembly and the DNA-BOT workflow. (**a**) BASIC DNA assembly workflow: Step 1: Clip reaction: simultaneous digestion and ligation attaches Prefix (P-) and Suffix (S-) half-linkers to parts. Step 2: Purification: clips are purified from the reactions via solid phase reversible immobilization (SPRI), removing excess linkers and enzymes. Step 3: Assembly: purified clips are annealed, forming circular constructs e.g. part A and B are annealed with a backbone (bb) part in a three-part assembly. Step 4: Transformation: assembled constructs are transformed into *E. coli*. (**b**) csv files describing source plates for parts, linkers and construct designs are processed by the DNA-BOT application (app), returning four OT-2 scripts along with meta-information files. Each script runs the corresponding BASIC step in microtiter plate format, finally spotting colonies on selective LB-agar plates. Dotted lines denote information flow and solid-lines denote physical processes.

The performance of automated cloning depends strongly on the accuracy and efficiency of the methodology in order to minimize the number of clones that need to be screened and potentially repeated. BASIC provides this high accuracy through the 21 bp single-stranded overhangs of linkers, which guide the assembly of linker-ligated and purified parts (Figure 2a), and high efficiency with simple, robust processes. Furthermore, the idempotent format enables hierarchical assembly through the exact same automation-friendly workflow, without incurring the problems associated with tiered assembly formats (4). These features make BASIC an ideal method for adaptation to an automated platform.

Currently, BASIC and alternative DNA assembly methods have only been automated using expensive infrastructure, limiting community access to the benefits that automated DNA assembly brings to research and applications in biology (5, 8–11). With the recent advent of the OT-2 liquid handling robot (Opentrons), equipment costs for entry-level automation dropped significantly, making it accessible to most molecular biology researchers. The OT-2 can accurately transfer volumes from 1 to 300 µl with single or 8-channel pipettes and supports BASIC integration through additional modules for automated temperature control and magnetic bead manipulation. A further advantage is the open source, python-based application programming interface that facilitates rapid protocol development.

Here, we present the DNA-BOT platform, which combines highly accurate, open-source BASIC DNA assembly with the low-cost Opentrons OT-2 for the automated assembly of genetic constructs. We hypothesized that DNA-BOT would be affordable for most research groups, while achieving the accuracy needed for large-scale, automated projects. Not only would this add to the available SynBioStack, it would improve the community’s ability to iterate through Design-Build-Test-Learn cycles, driving the development of Synthetic Biology.

## 2. Materials and methods

### 2.1 BASIC DNA parts and linkers

The fluorescent reporter proteins super-folder GFP (sfGFP) (12), mCherry (13) and an mTagBFP variant (BFP) (14), were all synthesized by TWIST (www.twistbioscience.com) flanked by BASIC Prefix and Suffix sequences. These DNA parts were cloned into an Amp-pUC storage vector that lacked BsaI sites (BASIC_SEVA_18_AmpR-pUC.1) via a two-part BASIC reaction, replacing a dropout mScarlet counter-selection cassette in the storage vector. The resulting plasmids and BASIC_SEVA_37_CmR-p15A.1 were prepared at the required scale using GenScript^®^’s Plasmid DNA Prep Service and diluted to 200 ng/µl ready to use in clip reactions.

A control plasmid based on BASIC_SEVA_37_CmR-p15A.1 that lacked the mScarlet counter-selection cassette was generated by first digesting BASIC_SEVA_37_CmR-p15A.1 with BsaI (NEB R0535). The linearized vector was blunted using the CloneJET PCR Cloning kit (Thermo Scientific K1232) according to the manufacturer’s instructions with 4 ng digested DNA. This was re-circularized by adding 1 µl each ddH_2_O and T4 DNA Ligase (Promega M1801) and incubating at 22°C for 30 min. Using this plasmid, DH5α *Escherichia coli* were transformed and white colonies lacking the mScarlet dropout cassette were selected on LB-agar plates supplemented with 25 µg/ml chloramphenicol. Plasmid DNA from these constructs was prepped using Omega BIO-TEK E.Z.N.A.^®^ Plasmid Mini Kit II. The desired plasmid was confirmed via Sanger Sequencing and by diagnostic digest using NgoMIV (NEB R0564) (data not shown).

A collection of neutral and functional linkers encoding RBS sequences or fusion peptides was designed and are available in a ready to use 96-well plate format (www.biolegio.com). For this study the standard BASIC linker set (Biolegio: BBP-19100) was used.

All DNA sequences used and generated during this study are available as genbank files at https://github.com/BASIC-DNA-ASSEMBLY/DNA-BOT/tree/oup_synbio/sequences. Furthermore, all plasmids are available upon request following the completion of any necessary Materials Transfer Agreements or supporting documentation.

### 2.2 DNA-BOT script generation

A csv file describing each of the 88 constructs was generated along with csv files describing the Biolegio BASIC linker set, standard linkers and the BASIC DNA parts required (Supplementary Data—storch_et_al_constructs.csv; BIOLEGIO_BASIC_STD_SET.csv; part_plate_2_230419.csv). As described in DNA-BOT_instructions_v1.0.0 (Supplementary Data), these csv files were used to generate four Opentrons OT-2 scripts. The fourth script was modified to transform and spot plasmid backbone and no plasmid controls in wells A12–H12. Furthermore, a fifth script was generated separately to spot 10 µl of each transformation reaction, as opposed to the 5 µl spotted during execution of the fourth script. All scripts used in this manuscript are available online (https://github.com/BASIC-DNA-ASSEMBLY/DNA-BOT/tree/oup_synbio).

### 2.3 DNA-BOT execution: DNA assembly

DNA-BOT was executed as described in detail within the Supplementary Data (DNA-BOT_instructions_v1.0.0). Compared to previous implementations of BASIC DNA assembly (4, 15), the following alterations were made: a clip reaction master mix was prepared manually by combining 3 μl of 10× Promega T4 DNA ligase buffer, 1 µl NEB BsaI-HF^®^v2 (R3733), 0.5 µl Promega T4 DNA Ligase (M1804) per 20 µl required for each reaction. The Opentrons OT-2 (www.opentrons.com) pipetted 20 μl of master mix for each reaction, plus Biolegio BASIC linkers and DNA parts, together with sufficient H_2_O to give a total volume of 30 µl. Clip reactions were incubated in a peqSTAR 96X Universal Gradient thermocycler for 20 cycles (37°C for 2 min, 20°C for 1 min), followed by a 5-min incubation at 60°C. For clip reaction purification 54 µl of AMPure XP magnetic beads (Beckman Coulter) were added; 150 µl 70% ethanol was used during wash steps; following resuspension in H_2_O, 38 µl of Elutant was transferred to a fresh well. Constructs were assembled in volumes of 15 µl using 1.5 µl of each purified clip reaction in a solution of 20 mM Tris: HCl (pH 8.0), 10 mM MgCl_2_, 50 mM KCl. Assemblies were incubated at 50°C for 45 min in a peqSTAR 96X Universal Gradient thermocycler. NEB^®^ 5-alpha Competent *E. coli*, 96 well plates (C2987P) were used for transformation reactions. Heat shocks were conducted according to the manufacturer’s instructions. SOC media (125 µl) was transferred to each assembly and the reaction incubated for 1 h with lids off at 37°C. Transformation reactions were spotted on Thermo ScientificTM Nunc™ OmniTray™ Single-Well Plate, non-treated (242811) plates containing 40 mL LB-agar supplemented with 25 µg/ml chloramphenicol.

### 2.4 Flow cytometry

Individual colonies were picked from agar plates generated by DNA-BOT and 200 μl LB medium (ForMedium) supplemented with 25 µg/ml chloramphenicol inoculated in 96-well plates. Cultures were incubated overnight, shaking at 600 rpm at 30°C. Overnight cultures were diluted 200 times into 100 μl LB supplemented with 25 µg/ml chloramphenicol. Cultures were grown shaking at 30°C for 6 h and 2 μl off-sampled into 200 μl phosphate buffer saline supplemented with 2 mg/ml kanamycin. Samples were analyzed for sfGFP, BFP and mCherry fluorescence using an Attune NxT Flow Cytometer with all samples gated using the same forward and side scatter settings. Data were analyzed using FlowJo_V10 and subsequently processed as described in the main text.

## 3. Results

BASIC DNA assembly is performed in four separate steps (Figure 2a). These were implemented as four individual processes on the OT-2, each with a dedicated deck setup (Supplementary Figure S1) for the associated script (Figure 2b). Briefly, in the first step BASIC clips are created by digesting BASIC parts out of their storage vectors and simultaneously ligating linkers that define the assembly order, in a one-pot enzymatic ‘clip’ reaction (Step 1). The resulting clips are purified from un-ligated linkers using solid-phase reversible immobilization (SPRI) paramagnetic beads (Step 2). These purified clips have 21 base single-stranded overhangs, facilitating their assembly when incubated at an appropriate temperature in annealing buffer (Step 3). Subsequent transformation of assembled constructs and plating on selection media (Step 4) yields colonies for downstream assays and applications.

After developing the principles of the robotic protocols to implement the four BASIC steps, we created an open-source python application that provides a convenient interface to generate scripts and associated parameters for the assembly and transformation of up to 96 constructs using BASIC DNA plasmid parts and Biolegio BASIC Linkers. The DNA-BOT application reads csv files detailing construct designs and plates containing BASIC parts and linkers to be used in a given project. Following the acquisition of these parameters the designs are analyzed and parsed into the required clip reactions and assembly instructions, directing the generation of four specific OT-2 scripts, for each of the four steps outlined above, along with associated meta-information (Figure 2b and Supplementary Figure S2).

The script for Step 1 provides instructions for the OT-2 to setup the clip reactions (up to 48 individual clip reactions are possible) required for the specified assemblies (Script 1); the reaction is performed in an external thermocycler. In Step 2, the OT-2 magdeck module is used to purify raw clip reactions from the left half of the 96-well plate using SPRI beads, depositing purified clips in the right half of the plate (Script 2). In Step 3, the appropriate purified clips are combined in annealing buffer to assemble each of the specified constructs; annealing is then performed in an external thermocycler (Script 3). In Step 4, assembled constructs are mixed with competent cells on the OT-2 before heat-shock transformation using an external thermocycler. After recovery in SOC medium, liquid cultures of transformed cells are spotted on a selective LB-agar plate (Script 4). Script 4 takes advantage of the OT-2 temperature deck which enables transformation setup at 4°C and outgrowth at 37°C. During the execution of these four scripts, the Opentrons app will instruct the user to setup the OT-2 deck space as required (Supplementary Figure S1), while prompting a few manual actions e.g. heat shock. Additionally, meta-information guides users through the composition of the required Clip Reaction Master Mix and the location of specific reagents. In the presented version, DNA-BOT automates BASIC DNA assembly using only Opentrons equipment and an external thermocycler as hardware in standard lab settings (Supplementary Data: DNA_BOT_instructions_v1.0.0).

**Figure 3.**
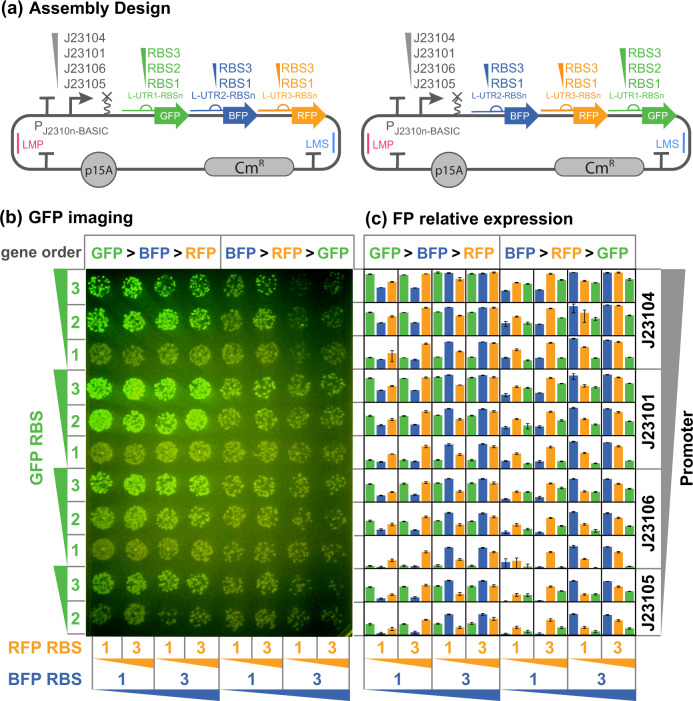
DNA-BOT provides for efficient and accurate DNA assembly. Relative promoter and RBS strengths are indicated by gradients. (**a**) Design of the 88 constructs assembled using DNA-BOT. The library contained full permutations of promoters and RBSs as indicated in two gene orders for the sfGFP (GFP), mCherry (RFP) and mTagBFP (BFP) genes, with the exception of the weak J23105 promoter and RBS1 GFP combination. (**b**) Image of the agar plate on a Safe Imager™ 2.0 Blue Light Transilluminator, acquired following the DNA-BOT workflow with 10 µl of each transformation reaction spotted. Operon design features for each well position are indicated at axes of panels b and **c**. Green colonies indicate strong sfGFP expression. (c) Green, orange and blue bars denote normalized mean superfolder GFP (GFP), mCherry (RFP) and mTagBFP (BFP) fluorescence, respectively, measured via flow cytometry for each construct from three biological repeats. Fluorescence is log scaled. Error bars denote standard deviations between three biological repeats.

To test DNA-BOT’s utility and ability to work at a relevant scale, we designed 88 constructs (Figure 3a) for assembly and transformation in parallel during a single run. Each variant encoded an operon expressing green, red and blue fluorescent proteins (sfGFP, mCherry and BFP) on a p15A backbone with a chloramphenicol-resistance cassette (Materials and methods). For these 88 constructs, 4 different promoters were used along with 2 or 3 different RBSs for each gene in 2 different gene orders; the 5′UTR and RBS for each gene was encoded on the linkers used to construct the operon, so these linkers were functional genetic components of the design. This design required 38 clip reactions to create the components for assembly of the final 88 constructs. In assembling these expression constructs, we benchmarked DNA-BOT’s performance while exploring an operon design space; one of many possible applications. Each construct consisted of five BASIC parts and five BASIC linkers with their identity defined for each variant in a construct design csv file. From this and csv files describing part and linker plates, the DNA-BOT application generated four scripts and meta-information for assembly and transformation (files available at https://github.com/BASIC-DNA-ASSEMBLY/DNA-BOT/tree/oup_synbio).

The workflow for the 88 assemblies was executed using the generated scripts and instructions, with the resulting transformants spotted in volumes of 5 µl and 10 µl onto SBS-LB-agar plates (Supplementary Figure S3, Materials and methods). Colonies were obtained for all 88 constructs and transformation control plasmids as expected (Figure 3b, Supplementary Figure S3). The transformants were analyzed for sfGFP fluorescence (Figure 3b) and triplicates for each assembly were picked for propagation in overnight liquid cultures. Overnight cultures were analyzed for sfGFP, mCherry and BFP fluorescence at the single cell level via flow cytometry (Figure 3c, Supplementary Figure S4, Materials and methods). These measurements enabled us to assess assembly success based on number and phenotypes of respective transformants across the 88 designs.

Observing the LB-agar plate in Figure 3b, we found each of the 88 spots contained a minimum of 5 colonies, returning transformants for all 88 constructs. Furthermore, cells exhibiting a pink phenotype were undetectable indicating a low background of un-digested backbone plasmid that would arise from expression of the mScarlet counter-selection marker. Colonies within each spot show a largely homogeneous sfGFP expression phenotype as one would expect if they carried the same expression construct.

Further characterization was performed by picking biological triplicates for flow cytometry (Materials and methods). Mean and standard deviations derived from background corrected and normalized geometric means were calculated; log-scale bar plots of the data are shown corresponding to the plate layout in Figure 3c. The flow data demonstrate the different genetic designs led to a diverse range of fluorescence outputs ranging over 4-orders of magnitude (Supplementary Figure S4). For each discrete design, we observed small standard deviations in the fluorescence response in almost all cases (Figure 3c and Supplementary Data: DNA_BOT_flow_data). Furthermore, the trends observed in the expression profiles of the three fluorescent reporters reflect the expected positive correlations between promoter strength, RBS strength, proximity to the start of the operon and expression strength, typically governing gene expression within operons (16). These observations indicate DNA-BOT performs DNA assembly with both high efficiency and high accuracy.

## 4. Discussion

DNA assembly is typically the starting point for Synthetic Biology projects and is therefore a critical technology for this field. The automation of DNA assembly methods facilitates larger more complex projects while increasing reliability and accuracy (17). While several reports have utilized automated DNA assembly methods (5, 9, 17, 18), they rely on expensive equipment which is often inaccessible for many research groups.

To reduce the barriers to entry and costs associated with automated DNA assembly, we developed DNA-BOT, a low-cost and open-source method. We validated DNA-BOT by assembling 88 constructs, each expressing a variant of a 3-gene operon. During this automated workflow, the OT-2 performed 1578 pipetting steps, 38 magnetic bead purifications and 96 heat-shock transformations in 96-well format. At the time of writing, the OT-2 including all required modules and pipettes costs around $8k. With a workflow starting from existing plasmid part libraries through to colony picking, the consumables cost per construct was as low as $1.50 or $5.50, depending on whether in-house or commercial competent cells are used, respectively (for a full description see Supplementary Table S1). This does not include sequence validation, but since the workflow starts from sequence verified plasmid parts and does not include PCR there is less imperative to sequence if it can be validated that all parts are present in the correct order, for instance by colony PCR (4). This compares favorably with similar platforms and strategies that have a significantly higher initial cost (17).

We estimated the operator hands-on time (not run time) for the automated process to be around 1 h 30 min, which compared favorably with well over 5 h when the same process was implemented manually, giving a Q_time_ metric (19) of 0.26 (Supplementary Table S2). While this illustrates considerable operator time saving, of greater significance is that the process is more robust and reliable, since robots typically outperform humans in repetitive tasks e.g. cherry-picking liquid transfers of small volumes.

To assess the efficiency and accuracy of DNA-BOT, we imaged agar plates yielded by the assembly method and selected three colonies for each construct to characterize sfGFP, mCherry and BFP expression following overnight growth at the single cell level using flow cytometry. On each agar plate, we observed colonies for all constructs, illustrating high efficiency. From both the largely homogeneous intensity of sfGFP for each assembly spot on the LB-agar plates and the small standard deviations observed from the flow cytometry measurements, we conclude that cells transformed with the same assembly have identical phenotypes, thus demonstrating that DNA-BOT provides high accuracy, in line with previous reports on the underlying assembly technology (4).

While the current performance of DNA-BOT is already very useful, we see several opportunities for future development. For instance, Opentrons will soon offer an onboard thermocycler for the OT-2, this will allow users to implement DNA-BOT relying on low-cost Opentrons hardware only, while potentially lowering the number of calibration steps. We will continue to develop the DNA-BOT software package to integrate with open-source DNA design tools like SBOL (20–22) and improve UX-design. Currently, two comprehensive BASIC linker sets are available ready to use on Opentrons in 96-well plates (Biolegio) and more BASIC parts will be made available, enriching the design opportunities for new BASIC and DNA-BOT users.

Until now, automated DNA assembly has largely been the preserve of well-funded institutions and Biofoundries. Here, we describe the implementation of BASIC DNA assembly on an open-source, low-cost automation platform. Our DNA-BOT package facilitates the generation of scripts to assemble and transform up to 96 BASIC constructs in a single run. The software tool abstracts away much of the molecular complexity of the build process, leaving the user free to focus on the biological design. The high accuracy and efficiency, together with the single-tier idempotent format derived from the underlying BASIC DNA assembly method (4), are convenient features at bench scale, but they become critical once DNA assembly is scaled in automated Design-Built-Test-Learn workflows (23). We are optimistic that DNA-BOT will make a significant contribution to the democratization of high-quality automated DNA assembly.

## Funding

UK Research and Innovation through the Engineering and Physical Sciences Research Council [EP/R034915/1]; the Biotechnology and Biological Sciences Research Council [BB/L027852/1] and EU H2020 [820699].

*Conflict of interest statement*. None declared.

## Supplementary Material

ysaa010_Supplementary_Data
