# Supplementary material for: DNA-BOT: a low-cost, automated DNA assembly platform for synthetic biology
Source: Synth Biol (Oxf). 2020 Jul 9;5(1):ysaa010. doi: 10.1093/synbio/ysaa010 (PMC7476404; doi:10.1093/synbio/ysaa010)
Supplement: ysaa010_Supplementary_Data [file ysaa010_supplementary_data.zip › DNA_BOT_instructions_v1.0.0.pdf]

Instructions for DNA-BOT (v1.0.0)

Contents

Contents 1
About 1
Materials 2
Methodology 4
  OT-2 preparation 4
  Construct and part/linker csv files 4
  Running the dnabot app 5
  Step 1: Clip reactions 6
    Preparation 6
    Script execution 6
    Thermocycler incubation 7
  Step 2: Purification 8
    Preparation 8
    Script execution 8
  Step 3: Assembly 9
    Preparation 9
    Script execution 9
    Thermocycler incubation 10
  Step 4: Transformation 11
    Preparation 11
    Script execution 11
  Colony generation and picking 13
  Validation and troubleshooting 13
Bibliography 14

About

This document is intended to guide users through DNA assembly using BASIC on OpenTrons (DNA-BOT).

Materials

Below we list the materials previously used to implement DNA-BOT. We recommend starting with these consumables, however certain materials like 96-well plates, microcentrifuge tubes etc. may be altered. Labware needs to be defined carefully when working on the Opentrons OT-2 and changes to labware require the user to update the template scripts[1] or those executed during DNA-BOT runs. We further advise users to follow general opentrons guidelines when utilising labware.

Software:

- Opentrons OT-2 Run App (Version 3.10.3 or later).
- dnabot GitHub repository for current software.
- Python 3 and packages specified in requirements.txt.

Hardware:

- Opentrons OT-2.
- Opentrons P10 Single-Channel Electronic Pipette.
- Opentrons P300 8-Channel Electronic Pipette.
- Opentrons Magnetic Module.
- Opentrons Temperature Module.
- Opentrons 4-in-1 Tube Rack Set.
- Opentrons Aluminum Block Set.
- Thermocycler compatible with 96-well plates.
- Microplate centrifuge.
- General laboratory equipment e.g. pipettes.

Consumables & reagents:

Item	Cat no./URL
Opentrons 10µL tips	https://shop.opentrons.com/products/opentrons-10ul-tips
Opentrons 300µL tips	https://shop.opentrons.com/collections/opentrons-tips/products/opentrons-300ul-tips
Framestar 96-well Rigid and Skirted PCR Plates	4ti-0960/RIG
Brooks Life Sciences Reservoir Plate, 21 mL channels	4ti-0131
Brooks Life Sciences 96 Square Deep Well Storage Microplate, 2.2 mL wells, U shaped bases	4ti-0136
Brooks Life Sciences PCR Foil Seals	4ti-0550

[1]Note changes to template scripts will apply to all implementations of the DNA-BOT app.

Thermo Scientific™ Nunc™ OmniTray™ Single-Well Plate, Non-treated	242811
1.5 mL STARLAB Crystal Clear Microcentrifuge tubes	E1415-1500
[optional] Brooks Life Sciences 96 Square Well Sealing Cap Mat	4ti-0137
Biolegio BASIC linker set	BBP-19100 https://www.biolegio.com/products-services/basic/
NEB BsaI-HF®v2	R3733
Promega T4 DNA Ligase	M1801
10x Assembly Buffer: 0.2 M Tris:HCl (pH 8.0), 0.1 M $MgCl_2$, 0.5 M KCl	N/A – generate from component materials (Sambrook and Russell 2001).
Beckman Coulter™ Agencourt AMPure XP SPRI paramagnetic beads	A6388
NEB® 5-alpha Competent E. coli, 96 well plate	C2987P

Additional reagents:

- ddH2O.
- Absolute ethanol.
- SOC media.
- LB-Agar + antibiotic e.g. chloramphenicol.

Methodology

OT-2 preparation

Follow the Opentrons guidelines for setting up the OT-2 before executing any protocols. 3rd-party labware not given in the default Opentrons OT-2 Labware Database but listed in the Materials section can be added to user OT-2 labware databases by running add_labware.ot2.py in the Opentrons app.

Construct and part/linker csv files

At least, two csv files are required to run the dnabot app, generating python scripts for the BASIC assembly on Opentrons OT-2. The first csv file contains the details of constructs to be assembled. Additional csv files are required for each 96-well plate containing BASIC parts and/or BASIC linkers that are used during clip reactions.

constructs_temp.csv provides a template to describe constructs for new DNA-BOT runs. As an example, storch_et_al_cons.csv describes constructs assembled in the OUP Synthetic Biology manuscript. **CONSTRUCTS MUST BE LISTED CONSECUTIVELY, WITH THE FIRST CONSTRUCT BEING ASSEMBLED IN WELL A1**. Each construct encompasses a single row of the csv file and is composed of alternating BASIC linkers and BASIC DNA parts. Each linker is defined by the overall linker ID. During assembly each linker is in fact ligated as half-linkers to different parts in separate clip reactions, after purification these then anneal to create the final construct. For instance, “LMS” refers to the sequence formed when the “LMS-S” and “LMS-P” half-linkers anneal during the assembly step of BASIC[2]. Any linker supplied by Biolegio can be used to design constructs[3].

parts_linkers_temp.csv provides a template to describe plates containing parts or linkers, used during clip reactions. Additionally, BIOLEGIO_BASIC_STD_SET.csv provides an example of a linker plate used to assemble constructs in the OUP Synthetic Biology manuscript. The first row of the csv file details the csv file header. The “Part/linker” column provides the ID of the part or linker. These exact ID values have to be used in the construct.csv file, with prefix and suffix linker IDs extended by “-P” and “-S”, respectively for the appropriate half-linkers. Parts and linkers not used to assemble constructs listed in the construct.csv file are ignored by the dnabot app during implementation. The “Well” column provides the corresponding well of the part or linker. No restrictions apply to well order and duplicating parts/linkers within the same file means only the latter record is used. This enables new aliquots of parts/linkers to be added to a plate in separate wells without having to remove the previous instance from the csv file. The “Part concentration (ng/uL)” column provides the corresponding concentration of DNA parts. 2.5 nM final concentration of DNA part during clip reactions is optimal (Storch et al. 2015) and given clip reaction volume and typical DNA part sizes (0.1-2kb) in storage vectors, 200 ng concentration for DNA parts is considered optimal by the dnabot app. BASIC DNA parts must be

[2] For additional understanding of the BASIC process, the original publication should be consulted(Storch et al. 2015).

[3] While additional, user-generated linkers are likely tolerated, they are currently discouraged.

supplied at a concentration > 25 ng/µL. For concentrations that are > 200 ng/µL and if this value is left blank, 1 µL is pipetted. Using concentrations higher than 200 ng/µL will not improve the efficiency of the process and is not recommended.

Running the dnabot app

To acquire the dnabot app, clone the dnabot GitHub repository. Note, the dnabot package is not intended to be installed at the environment level and is instead a stand-alone application. The simplest way to start the dnabot app is to run the dnabot_app.py module from within the dnabot package using an appropriate Python environment. This will bring up a simple graphic user interface with instructions. Follow these instructions to generate the 4 scripts and metainformation required to execute the DNA-BOT workflow. Following app execution, scripts and metainformation are located in the directory containing the construct csv file.

Step 1: Clip reactions

Preparation

Prepare PCR plates containing DNA parts and linkers, ensuring no air gaps exist at the bottom of wells and that sufficient material is available. A dead volume of 10 – 15 µL for each part and linker is required to ensure complete liquid transfers. For consistent results, DNA parts should be well mixed prior to script execution. Linkers stored for >1 month should be reannealed according to BioLegio instructions.

Prepare the clip reaction MASTER_MIX in a microcentrifuge tube by combining the required materials specified in the "[construct csv file name]_clip_run_info.csv" file[4], located in the "metainformation" directory. Prepare an additional microcentrifuge tube containing ddH$_2$O.

Script execution

Execute the "1_clip.ot2.py" script using the Opentrons app and follow the instructions for calibration and running. An example deck layout is given below. Plates containing parts and linkers must be placed in deck positions as specified in the "[construct csv file name]_clip_run_info.csv" file. Additionally, place tubes containing master mix and water in positions A1 and A2 of the tube rack, respectively. A fresh PCR plate should be placed on deck position 1 for clip reactions.

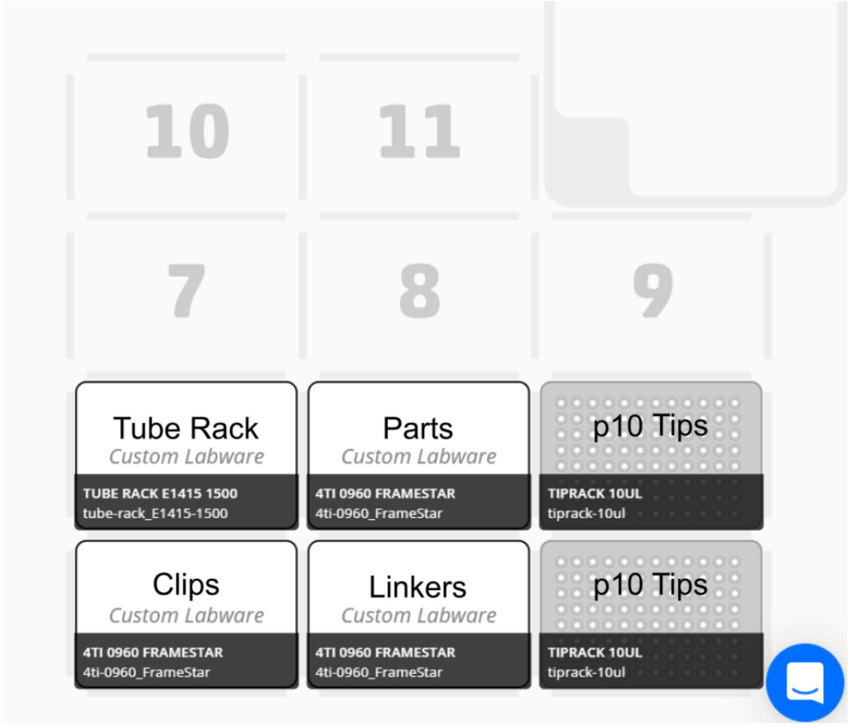

[4] Refer to storch_et_al_cons_clip_run_info.csv for a previously generated example.

Thermocycler incubation

Following completion of the “1_clip.ot2.py” script, remove the plate containing clip reactions from the OT-2. Seal the plate using a foil seal and transfer to a thermocycler. Conduct the following incubation:

Temperature	Time	
37°C	2 min	**X 20 cycles**
20°C	1 min	
60°C	5 min	
4°C	hold	

Step 2: Purification

Preparation

Following clip reaction incubation, remove the foil seal from the plate containing raw clip reactions. Prepare a trough with ddH_2O in well A1 and fresh 70 % EtOH[5] in the selected well[6]. Resuspend the SPRI beads (AMPure XP, Beckman Coulter) and aliquot 0.75 mL into wells A1 – H1, inclusive of a 96 deep-well plate[7].

Script execution

Execute the "2_purification.ot2.py" script using the app and follow the instructions for calibration and running. An example deck layout is given below. A 96 well plate, referred to as a "Mix Plate" is required for effective SPRI immobilisation.

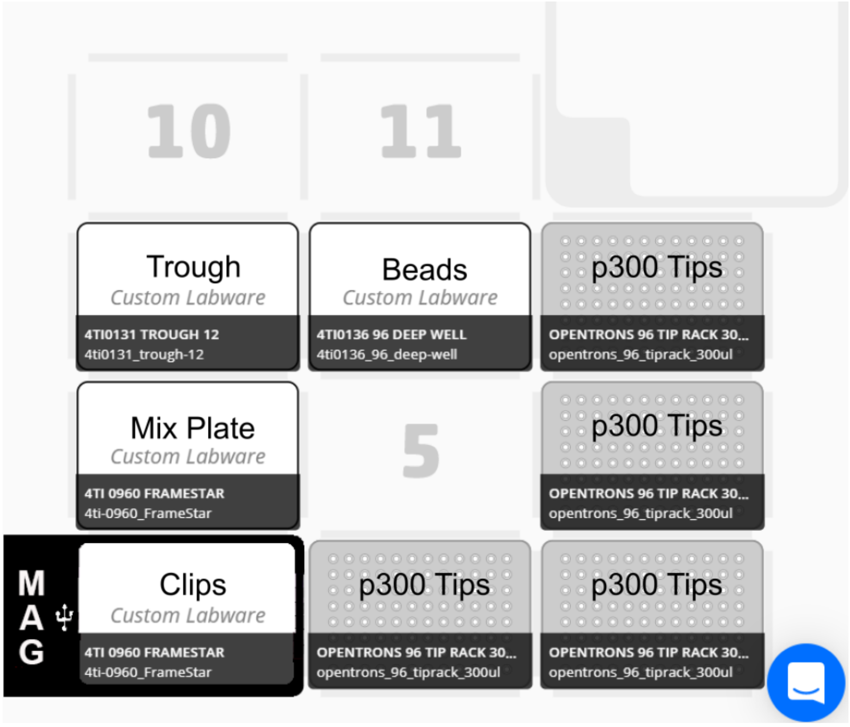

[5] Follow the SPRI bead manufacturers guidelines for the preparation of 70 % EtOH.
[6] If required, refer to the metainformation directory or "2_purification.ot2.py" script for the previously selected well.
[7] Beads can be stored within the 96 deep well plate using the corresponding seal mats. To resuspend within the plate simply invert several times before removing the seal. Ensure sufficient bead volume is available in each well before executing scripts.

Step 3: Assembly

Preparation

Optionally seal the plate containing purified clips with a foil seal and centrifuge to guarantee the absence of air gaps in wells. Air gaps in wells can cause liquid transfers during assemblies to fail.

Assemblies consisting of a different number of parts, require different dilutions of 10x Assembly Buffer (see table below). This functions to reduce the number of pipetting steps, saving time and resources. The trade-off is that multiple solutions must be generated when assembling constructs containing different part numbers. However, Assembly Buffer stores well at 4 °C meaning dilutions can be prepared in bulk (≥ 10 mL), aliquoted and stored until required (see table below).

Prepare the required dilutions of Assembly Buffer for the constructs being assembled in microcentrifuge tubes. Place the tube in the specified tube rack well (see table below).

Number of parts to assemble	[Assembly Buffer] (fold)	10x Assembly Buffer for 10 mL (mL)	ddH_2O for 10 mL (mL)	Suggested aliquot (mL)	Well in tube rack
2	1.3	1.3	8.7	1.25	A1
3	1.4	1.4	8.6	1.1	A2
4	1.7	1.7	8.3	0.95	A3
5	2	2	8	0.8	A4
6	2.5	2.5	7.5	0.65	A5

Script execution

Execute the “3_assembly.ot2.py” script using the app and follow the instructions for calibration and running. An example deck layout is given below. A fresh, 96-well PCR plate should be used for assemblies. This plate is placed on the aluminium 96-well block which in turn is placed on the Temperature Module.

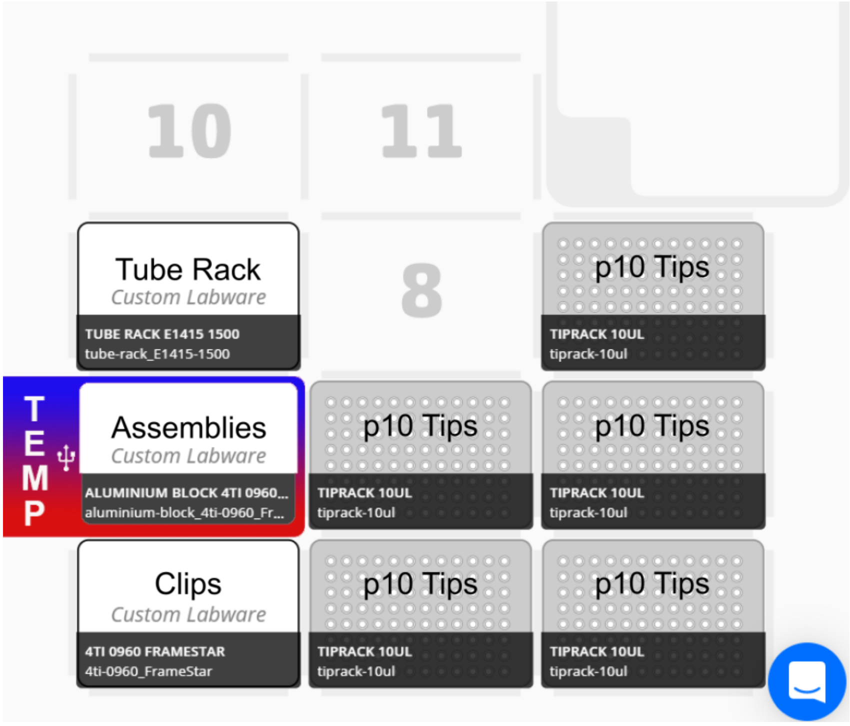

Thermocycler incubation

Following completion of the “3_assembly.ot2.py” script, remove the plate containing assemblies from the OT-2. Seal the plate using a foil seal and transfer to a thermocycler. Conduct the following incubation:

Temperature	Time
50°C	45 min
4°C	hold

Step 4: Transformation

Preparation

Remove the plate containing the assemblies from the thermocycler and remove the foil seal.

Prepare a plate for SOC media by first sterilising a 96 deep-well plate and then filling each well of the selected column[8] with 2 mL sterile SOC media. Avoid touching neighbouring wells, enabling remaining columns to be used during subsequent runs.

Prepare the agar plate by pouring 40 mL LB-agar supplemented with the required concentration of antibiotic/s into a Nunc™ Omnitray™. Leave to set, and dry prior to use[9].

Place the aluminium 96-well block on ice or at 4 °C to chill. Place the plate containing competent cells on the chilled block, remove the foil and cover with the provided plate cover. Place the block with cells on ice until required.

Script execution

Execute the "4_transformation.ot2.py" script using the app and follow the initial instructions for pipette calibration. An example deck layout is given below. Continue the calibration protocol, calibrating pipette tips. To calibrate well A1 of the agar plate, first calibrate the x and y-axes using a 96-well PCR plate. Remove the PCR plate and reintroduce the agar plate to calibrate the z-axis, move the pipette tip until it just touches the agar. Continue with the instructions to calibrate the remaining labware. The tube rack requires a single empty microcentrifuge tube in well A1 for spotting waste. Once the plate containing cells has been calibrated, cover and store on ice until prompted to return[10].

Proceed by running the protocol. The script will first cool the temperature deck to 4 °C, before pausing, allowing users to place competent cells on the deck. After resuming, the OT-2 will mix assemblies with competent cells and incubate for 20 minutes. The user is then prompted to conduct a heat shock. To achieve this, preheat a thermocycler to 42 °C and remove cells to ice. Heat shock at 42 °C for 15 seconds in the thermocycler and return to ice for 2 minutes. Transfer the cells back to the temperature deck and resume the run. No further pauses are implemented, allowing users to return after script completion.

---

[8] If required, refer to the metainformation directory or "4_transformation.ot2.py" script for the previously selected column of wells.

[9] To avoid spots fusing during spotting, agar plates must be well dried. Previously, plates have successfully been dried at 37 °C for 3 – 4 hours.

[10] The agar plate and SOC media can also be removed from the deck and covered until after heat shock, to reduce the risk of contamination.

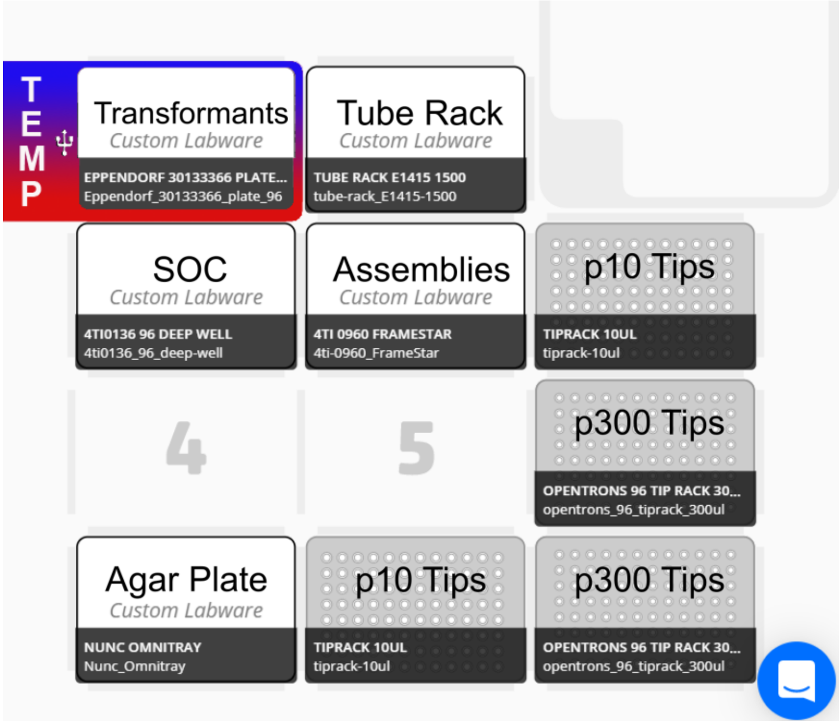
TEMP
Transformants
Custom Labware
EPPENDORF 30133366 PLATE...
Eppendorf_30133366_plate_96
Tube Rack
Custom Labware
TUBE RACK E1415 1500
tube-rack_E1415-1500
SOC
Custom Labware
4TI0136 96 DEEP WELL
4ti0136_96_deep-well
Assemblies
Custom Labware
4TI 0960 FRAMESTAR
4ti-0960_FrameStar
p10 Tips
TIPRACK 10UL
tiprack-10ul
4
5
p300 Tips
OPENTRONS 96 TIP RACK 30...
opentrons_96_tiprack_300ul
Agar Plate
Custom Labware
NUNC OMNITRAY
Nunc_Omnitray
p10 Tips
TIPRACK 10UL
tiprack-10ul
p300 Tips
OPENTRONS 96 TIP RACK 30...
opentrons_96_tiprack_300ul

Colony generation and picking

Following spotting, incubate agar plates at 37 °C until colony formation and apply a foil seal to the plate containing transformation reactions. Transformation reactions can be stored as backup at 4 °C for several days.

To isolate single colonies for downstream applications users may be required to conduct further manual steps. When no colonies form following spotting, the remaining transformation reaction should be plated on an agar plate (Sambrook and Russell 2001). This facilitates the screening of larger volumes of transformation reaction for the presence of successful transformants. In addition to observing no colonies, it is possible that colony density is too high for single-colony isolation. This is likely to be the case for assemblies composed of only 2 or 3 parts, where the transformation efficiency is higher. To overcome this, spots with high colony densities should be streaked-out (Sambrook and Russell 2001).

Validation and troubleshooting

The validation of BASIC DNA assembly constructs has previously been achieved using colony PCR with primers that anneal to linker sequences (Storch et al. 2015). The same method can be applied to validate DNA-BOT constructs and it can enable rapid and accurate determination that all parts are present in the correct order. For the analysis of many constructs in parallel, fragment analyser or tapestation systems (e.g. Agilent Large Fragment Kit: DNF-492-0500) may provide a scalable alternative to agarose electrophoresis.

If colonies are not detected following manual plating, two measures should be taken to identify and alleviate causes of failure. Firstly, raw and purified clip reactions can be analysed using either agarose electrophoresis (Storch et al. 2017) or fragment analyser systems. Agarose gel electrophoresis can determine if parts have been completely digested from storage vectors and whether an acceptable yield has been achieved following purification: complete digestion of the BASIC DNA part and > 60 % yield following purification are required for effective DNA-BOT workflows. Equivalent BASIC DNA parts digested with *BsaI* can function as a positive control for this step. Fragment analysers can, for parts <2kb, provide sufficient resolution to discriminate between the digested part and the linker adducted part, so it is possible to determine directly the efficiency of the clip reaction. As a second step, users should test the quality of their competent cells. It has previously been reported that BASIC DNA assembly requires highly competent cells of 10^9 CFU/µg pUC19 for the assembly of up to seven parts (Storch et al. 2017).

It is good practice to include control assemblies with known DNA parts to ensure that the workflow is functioning. If there are repeated failures of specific designs then the Biodesign Engineer should consider whether their genetic design is not within the operational envelope of their chassis. This may be caused by constructs with strong promoters, strong RBSs and high copy number causing a translational burden on cells. Alternatively, specific genes or gene products may be toxic. Designs should be modified to reduce translational stress and potentially even remove promoters from specific genes to evaluate if their expression leads to toxicity.

Bibliography

Sambrook, Joseph, and David Russell. 2001. *Molecular Cloning A Laboratory Manual*. 3rd ed. New York: Cold Spring Harbor Laboratory Press.

Storch, Marko, Arturo Casini, Ben Mackrow, Tom Ellis, and Geoff S. Baldwin. 2017. “BASIC: A Simple and Accurate Modular DNA Assembly Method.” *Synthetic DNA: Methods and Protocols, Methods in Molecular Biology* 1472: 35–45. https://doi.org/10.1007/978-1-4939-6343-0.

Storch, Marko, Arturo Casini, Ben Mackrow, Toni Fleming, Harry Trewhitt, Tom Ellis, and Geoff S. Baldwin. 2015. “BASIC: A New Biopart Assembly Standard for Idempotent Cloning Provides Accurate, Single-Tier DNA Assembly for Synthetic Biology.” *ACS Synthetic Biology* 4 (7): 781–87. https://doi.org/10.1021/sb500356d.
