# Supplementary material for: DNA-BOT: a low-cost, automated DNA assembly platform for synthetic biology
Source: Synth Biol (Oxf). 2020 Jul 9;5(1):ysaa010. doi: 10.1093/synbio/ysaa010 (PMC7476404; doi:10.1093/synbio/ysaa010)
Supplement: ysaa010_Supplementary_Data [file ysaa010_supplementary_data.zip › Storch_et_al_2019_Supplementary_OUP.pdf]

Supplementary Data

DNA-BOT: A low-cost, automated DNA assembly platform for synthetic biology, Storch et al. 2019

Supplementary figures 2
Supplementary tables 6
Bibliography 8

Supplementary figures

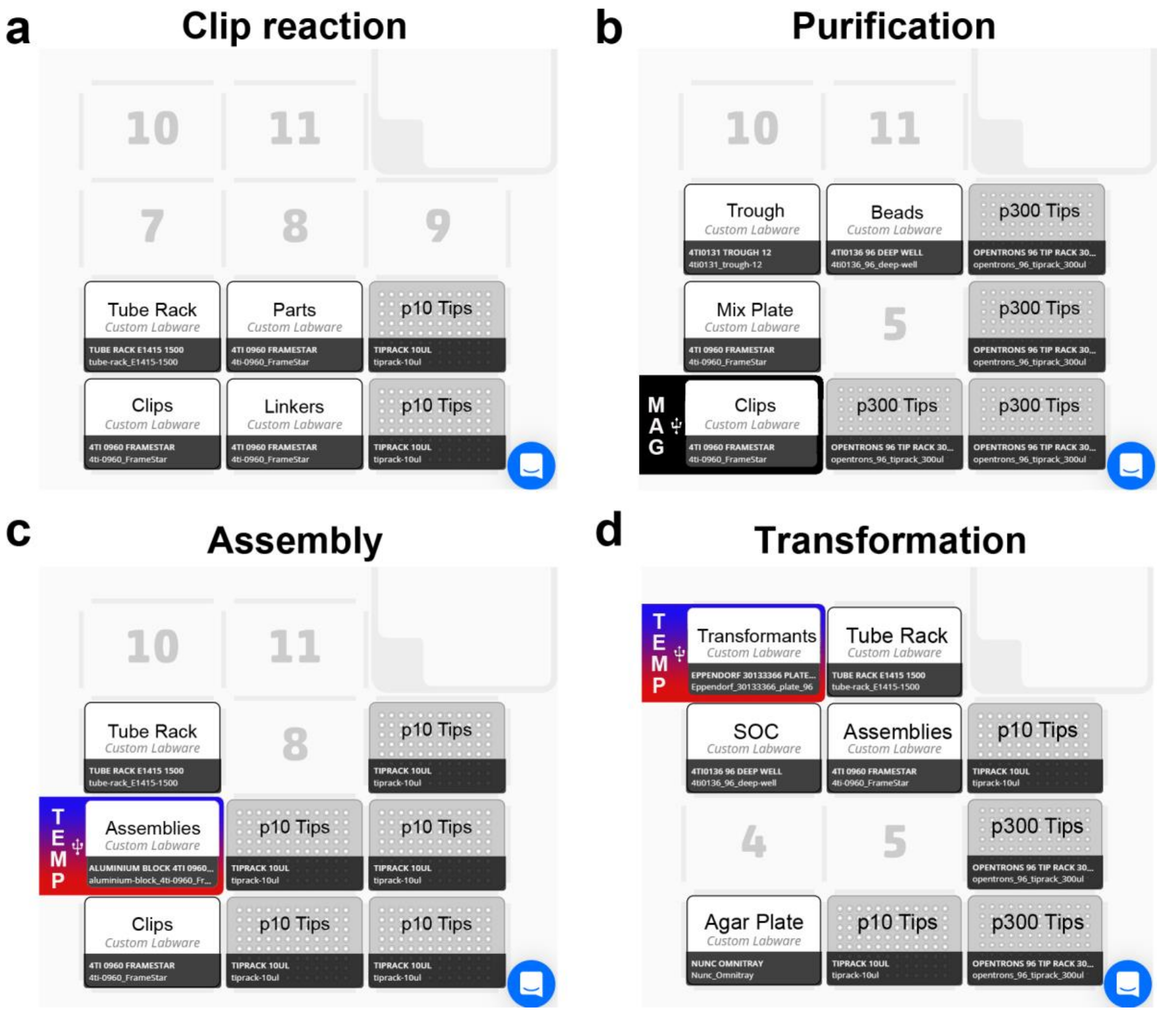

Figure S1. Deck layout used during generation of 88 constructs using DNA BOT (a) Script 1: Clip reaction, (b) Script 2: Purification, (c) Script 3: Assembly and (d) Script 4: Transformation.

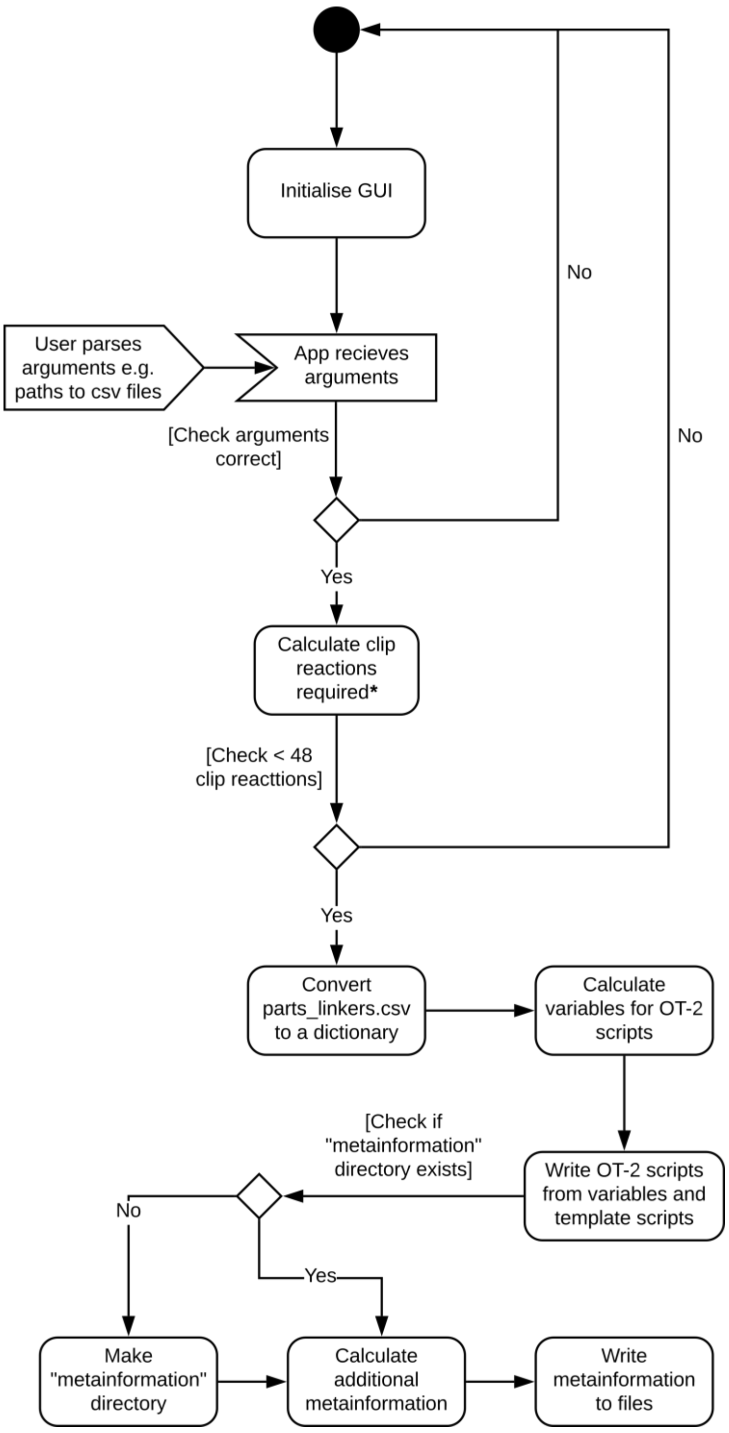

Figure S2 UML activity diagram[1] of the DNA-BOT application indicated in Figure 2b. For each activity, the corresponding comment within the main() function of dnabot_app.py indicates the relevant lines of code. ***During** the “calculate clip reactions required” step, the required clips (part and half-linker combinations) are determined for all part and linker combinations associated with multiple constructs across the specified designs. Where the same clip reaction is used in multiple assemblies (e.g. backbone vector), the total number of clip reactions required is calculated based on each purified clip being sufficient for up to 15 different assemblies.

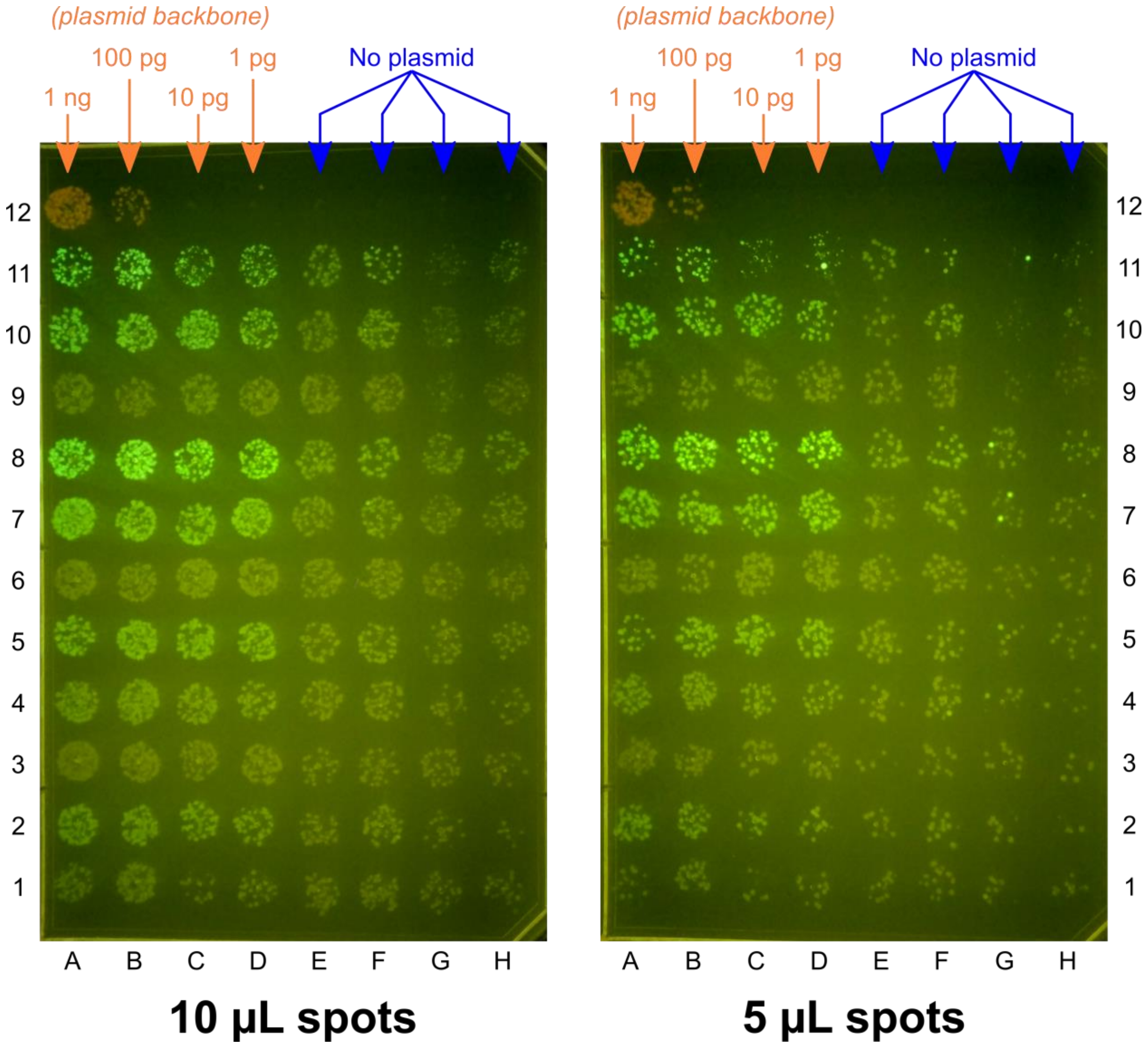

Figure S3. Agar plates imaged for GFP on a Safe Imager™ 2.0 Blue Light Transilluminator. Plates were spotted with 10 or 5 µL of each transformation reaction. Corresponding well identities can be inferred by the surrounding grid of letters and numbers. Cells spotted on positions A12 – H12, were transformed with 1 ng, 100 pg, 10 pg, 1 pg of BASIC_SEVA_37_CmR-p15A.1 (plasmid backbone with mScarlet counter selection cassette) or with plasmid-free H_2O (no plasmid), respectively. (Materials and methods).

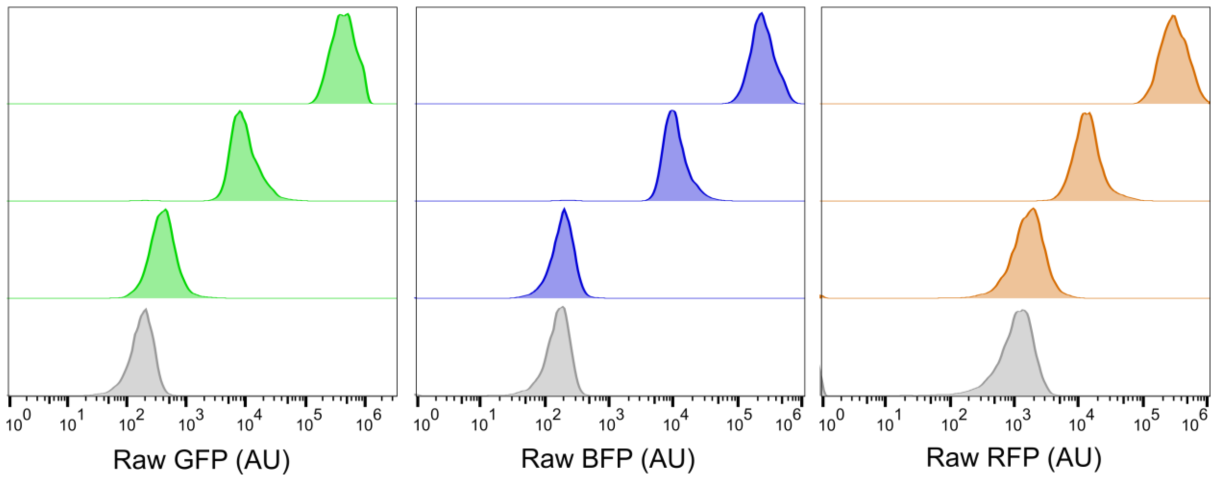

Figure S4. Flow cytometry histograms illustrating dynamic range in fluorescent reporter expression strength. Negative control cells with empty backbone are shown in grey and examples for low, medium and high expression phenotypes from the 88 construct library are shown in green (GFP), blue (BFP) and orange (RFP). Specifically, data for the following constructs are shown in order of low, medium and high expression, respectively: GFP: G3, H5 and C11. BFP expression: E2, H1 and H10. RFP: A1, F4 and H10.

Supplementary tables

Table S1: DNA-BOT running costs per construct

Costing assumes constructs are composed of 5 parts and each purified clip is used in 15 assemblies, representative for the 88 constructs. Costs for foil seals, linkers, trough, microcentrifuge tubes, assembly buffer, ethanol and water were considered neglectable. Unit prices correct as of October 2019 and values given to 2 DP.

**Item**	**Units**	**Units per construct**	**Unit price (USD)**	**Cost (USD)**
Opentrons p10 tips	tips	9.33	0.03	0.27
Framestar 96-well Rigid and Skirted PCR Plates	plates	0.02	3.69	0.06
Promega T4 DNA Ligase	µL	0.17	1.18	0.20
NEB BsaI-HF®v2	µL	0.33	1.11	0.37
Opentrons p300 tips	tips	5.00	0.03	0.14
Brooks Life Sciences 96 Square Deep Well Storage Microplate	plates	0.01	3.69	0.04
Beckman Coulter™ Agencourt AMPure XP SPRI paramagnetic beads	mL	0.02	16.62	0.30
NEB® 5-alpha Competent E. coli, 96 well plate	*plate*	*0.01*	*380.12*	*3.96*
Thermo ScientificTM Nunc™ OmniTray™ Single-Well Plate	plate	0.01	3.58	0.04
**Total**				**5.37**
**Total – w/o competent cells**				**1.41**

Table S2: Q-metric of hands-on time required for manual BASIC assembly and DNA-BOT. Calculated for the implementation of 48 clip reactions, plus the assembly and transformation of 88 constructs. The Q_{time} metric[2] has been calculated from these two values.

Step	Manual protocol (mins)	Automated protocol (mins)
Clip reactions (48)	90	15
Purification	30	25
Assembly	120	15
Transformation	100	35
**Total**	**340**	**90**
**Q_{Time} = 0.26**		

Bibliography

(1) Fowler Martin, 1963-. *UML Distilled : A Brief Guide to the Standard Object Modeling Language /*, 3rd ed.; Addison-Wesley: Boston, MA ;

(2) Walsh, D. I.; Pavan, M.; Ortiz, L.; Wick, S.; Bobrow, J.; Guido, N. J.; Leinicke, S.; Fu, D.; Pandit, S.; Qin, L.; Carr, P. A.; Densmore, D. Standardizing Automated DNA Assembly: Best Practices, Metrics, and Protocols Using Robots. *SLAS Technol. Transl. Life Sci. Innov.* **2019**, 247263031882533. https://doi.org/10.1177/2472630318825335.
